# Seroprevalence of *Toxoplasma gondii* in Poultry from Backyard and Intensive Production Systems in Romania

**DOI:** 10.3390/ani16152313

**Published:** 2026-07-27

**Authors:** Maria Nedișan, Adriana Györke, Vlad-Dan Cotuțiu, Mircea Coroian, Toma Naic Andra, Vasile Cozma, Radu Blaga, Amandine Blaizot, Isabelle Villena, Călin Gherman

**Affiliations:** 1 Department of Parasitology and Parasitic Diseases, Faculty of Veterinary Medicine, University of Agricultural Sciences and Veterinary Medicine Cluj-Napoca, 3-5 Calea Mănăştur Street, 400372 Cluj-Napoca, Romania; maria.nedisan@usamvcluj.ro (M.N.); adriana.gyorke@usamvcluj.ro (A.G.); andranaic@gmail.com (T.N.A.); vasile.cozma@usamvcluj.ro (V.C.); calin.gherman@usamvcluj.ro (C.G.); 2 AINIMAL SRL, Strada Stejarului 41D, 407280 Florești, Romania; vlad.cotutiu@usamvcluj.ro; 3 Department of Poultry Management and Pathology, University of Agricultural Sciences and Veterinary Medicine of Cluj-Napoca, 400372 Cluj-Napoca, Romania; 4 Anses, INRAE, Ecole Nationale Vétérinaire d’Alfort, Laboratoire de Santé Animale, BIPAR, 94700 Maisons-Alfort, France; radu.blaga@vet-alfort.fr (R.B.); amandine.blaizot@anses.fr (A.B.); 5 UR ESCAPE, Université Champagne-Ardenne, 51100 Reims, France; ivillena@chu-reims.fr; 6 Centre National de Référence Toxoplasmose, Centre Hospitalier Universitaire, 51092 Reims, France

**Keywords:** *Toxoplasma gondii*, poultry, Romania, Modified Agglutination Test, seroprevalence, backyard production, intensive poultry systems, One Health

## Abstract

*Toxoplasma gondii* is a cosmopolitan zoonotic parasite for which free-range poultry can act as sentinels of oocyst contamination. Romanian avian data on toxoplasmosis are limited to extensive systems and wildlife, while poultry seropositivity in intensive systems has not been previously characterized. In this study, 1940 domestic birds from intensive and extensive production systems were tested for *T. gondii* IgG antibodies by the Modified Agglutination Test (MAT). The descriptive dataset included data on 813 intensive broiler chickens and 1127 backyard birds. Backyard chickens had roughly twice the MAT seroprevalence of intensive broilers (31.2% vs. 14.1%, *p* < 0.001), rising to more than a tenfold difference at ≥1:48 (18.7% vs. 1.7%, *p* < 0.001). Seroprevalence varied among counties and farms, but intensive broilers were sampled in a very delimited area, so geographic and production-system patterns partly overlapped and should not be interpreted independently. In models adjusting for county and age, intensive production remained associated with lower odds of infection. These findings establish the first Romanian seroprevalence baseline for intensive poultry and support risk-based surveillance focused on backyard flocks and counties with high extensive-system prevalence.

## Simple Summary

*Toxoplasma gondii* is a parasite that can infect people and animals and is often linked to cats and contaminated soil. Free-range poultry can show whether this parasite is circulating in the local environment through their ground foraging behavior. In Romania, backyard flocks are common, while industrial poultry are raised under stricter biosecurity, yet little is known about infection in both systems together. This study tested blood from backyard and industrial chickens for antibodies against *T. gondii*, which show past exposure to the parasite rather than current infective status. Backyard chickens were more often exposed than industrial birds, but industrial flocks were not free of exposure. Higher exposure in backyard birds supports their use as practical sentinels of environmental contamination. For society, this work helps explain why rural backyard poultry matter for One Health. They can flag local environmental contamination and remind households and producers that reducing contact with contaminated soil and practicing safe food handling remain important, even when commercial biosecurity reduces, but does not remove, risk.

## 1. Introduction

*Toxoplasma gondii* is a cosmopolitan zoonotic apicomplexan protozoan capable of infecting all warm-blooded animals [1,2,3]. Its life cycle centers on felids as the sole definitive hosts, which shed environmentally resistant oocysts in their feces, while intermediate hosts harbor tissue cysts primarily in muscle and neural tissue [4,5]. Toxoplasmosis represents a public health concern; human infection occurs predominantly through consumption of undercooked meat containing tissue cysts or through ingestion of food and water contaminated with sporulated oocysts from the environment [2]. Primary infection during pregnancy can cause severe congenital disease, and reactivation in immunocompromised patients can be fatal [3,6].

Domestic poultry are of particular epidemiological interest in the context of *T. gondii*, as backyard and free-range flocks serve as sentinels of environmental oocyst contamination through ground foraging within a limited home range [2,7]. Seropositivity in these birds can further indicate settings where human exposure to infected poultry tissues may be plausible, particularly where slaughter and consumption occur outside regulated chains, although serology alone cannot quantify human risk [2]. In Brazilian smallholdings, high seroprevalence in free-range chickens was accompanied by the isolation of multiple *T. gondii* strains with considerable genotypic diversity, supporting the chickens’ use as sentinels of active environmental circulation as well as potential exposure pathways [8,9]. The seroprevalence of *T. gondii* in poultry varies greatly depending on the production system: values approaching 0% have been reported in broiler chickens raised under strict indoor confinement, while backyard and organic free-range flocks may reach 100% seropositivity [2].

In Europe, German backyard laying hens reached 47.7% seroprevalence versus negligible infection on large organic farms [10]. In Greece, 41.2% of backyard chickens were infected, with zero seropositive birds among indoor-housed intensively reared flocks [11]. In Spain, Australia, China, Libya, Brazil and Sub-Saharan Africa, *T. gondii* DNA was detected in ~10% of retail free-range chicken meat [7,12,13,14,15,16,17,18,19]. Global meta-analyses estimated the pooled avian seroprevalence at approximately 24% [20], with intensive systems rarely exceeding 5–8%, while backyard flocks range within 20–50% [21].

Beyond their sentinel role, domestic birds represent a direct zoonotic risk. Poultry meat is among the most widely consumed animal proteins globally, and *T. gondii* tissue cysts persist in edible tissues of seropositive birds [2,4], although due to their size they are not detected by routine macroscopic meat inspection [2,22]. In rural settings where birds are slaughtered informally at the household level or outside regulated slaughterhouse surveillance, seropositive poultry represent a plausible but unquantified exposure route linked to private/local consumption practices rather than to a failure of official veterinary inspection per se [23].

In Romania, human *T. gondii* seroprevalence ranges from 36% to 70% depending on the county, age group, and diagnostic method [24], indicating widespread exposure. Despite this, the epidemiology of avian toxoplasmosis in the country has received comparatively little attention. Nedisan et al. [25] reported 38.6% seroprevalence in backyard domestic birds using ELISA, identifying cat and mouse presence, age above one year, and outdoor access as risk factors. More recently, Gherman et al. [26] documented 19.5% seroprevalence in wild corvids using MAT, with all genotyped isolates belonging to Type III/Clade B, consistent with the broader European avian context [6,27].

Romania’s agricultural landscape is characterized by a dual production structure: a large-scale industrial poultry sector operating under European Union biosecurity standards alongside a widespread traditional backyard farming sector, particularly in rural areas where cats are ubiquitous [24]. This coexistence provides a natural framework for investigating how production systems mediate *T. gondii* exposure, and whether industrial biosecurity in the Romanian context provides effective protection against infection.

The present study, therefore, investigated anti-*T. gondii* antibodies by the Modified Agglutination Test in domestic birds from Romanian intensive and extensive production systems. The analysis was designed to estimate overall and system-specific seroprevalence, retain other species as a descriptive context, and focus formal inference on the chicken-only dataset. Within this framework, the study assessed production system, geography, age, and household variables; examined the dilution profile across MAT thresholds; and used regularized logistic regression to summarize adjusted associations. These analyses provide the first structured comparison of extensive and intensive Romanian poultry systems and establish a baseline for future targeted surveillance and molecular follow-up.

## 2. Materials and Methods

### 2.1. Study Design and Sampling

This cross-sectional epidemiological study investigated *T. gondii* seroprevalence in domestic birds from Romania across two distinct production systems, between 2017 and 2019. Blood samples were collected from the ulnar (wing) vein for backyard birds. Serum was separated from clots by centrifugation and stored at −25 °C until testing. Sample size calculations for backyard chickens assumed an expected prevalence of ~50% (95% confidence level) as no robust prior prevalence estimate was available and this assumption provides the most conservative sample size. For intensive broilers, an expected prevalence of 10% was assumed based on the lower seroprevalence reported in commercial poultry in previous studies. The minimum required sample sizes were 385 and 139 birds to attempt to infer epidemiological and statistical relevance.

#### 2.1.1. Extensive (Backyard) System

During 2017–2018, serum samples were collected from 1127 free-range domestic birds comprising five species, chicken (*n* = 1067), turkey (*n* = 27), duck (*n* = 26), guinea fowl (*n* = 5), and pigeon (*n* = 2), across 38 localities in 12 counties of Romania (Argeș, Bihor, Bistrița-Năsăud, Brașov, Cluj, Covasna, Maramureș, Salaj, Satu Mare, Tulcea, Vaslui and Vâlcea). Backyard counties and localities followed the USAMV Cluj extensive surveillance program previously reported by Nedișan et al. [25] and were expanded in the present MAT cohort. The ages of the sampled birds ranged from 24 to 156+ weeks. Eight to ten birds were sampled per household. For each bird, the following variables were recorded via a questionnaire administered to the owners: species, age (weeks), locality, county, presence or absence of cats and mice at the household, and flock size. Samples were stored at 2 °C for transportation.

#### 2.1.2. Intensive (Industrial) System

Between April and September 2019, serum samples were collected from 813 broiler chickens of slaughter age (42 days) at three industrial poultry farms located in three counties (Alba, Mureș, Sibiu) during commercial slaughter. Farms were selected based on slaughterhouse access and established collaboration with operators, while being adjacent to counties in the previously mentioned extensive system study [25].

### 2.2. Modified Agglutination Test (MAT)

*Toxoplasma gondii*-specific IgG antibodies were detected by the Modified Agglutination Test (MAT) as described by Desmonts and Remington [28]. The MAT was selected because the extensive survey included multiple backyard avian species (chickens, turkeys, ducks, guinea fowl, and pigeons) in addition to broiler chickens; unlike ELISA or IFAT, the MAT does not require species-specific secondary antibodies and therefore allowed a single serological panel across the full sample set [29,30]. Formalin-fixed whole RH-strain tachyzoites were supplied by the National Reference Centre for Toxoplasmosis, Laboratory of Parasitology, University of Reims Champagne-Ardenne, Reims, France [31], where the assay is routinely applied and validated in avian hosts.

Sera from the backyard poultry were tested at four serial dilutions: 1:6, 1:12, 1:24, and 1:48. Those collected from broiler chickens were tested at dilutions 1:24 and 1:48. In intensive broilers, reactivity at 1:6 and 1:12 was inferred from directly measured 1:24 results using the MAT serial-dilution cascade (positivity at 1:24 implies positivity at lower dilutions), validated with 100% consistency in the extensive dataset (Table S2). Positive and negative control sera were included on each plate. A positive result was defined as visible agglutination covering at least 50% of the well diameter after 12 h of incubation at room temperature. Sera reactive at any tested dilution were classified as MAT-positive for descriptive reporting, while the 1:24 threshold was used as the common cut-off for comparisons between production systems.

### 2.3. Statistical Analysis

All analyses were performed in Python 3.14 using scipy (v 1.17.0), stats models (v 0.14.6), scikit-learn (v 1.8.0), and pandas (v 3.0.0). Statistical significance was set at α = 0.05, with Bonferroni correction applied for multiple comparisons. Due to the low sample size, bivariate and multivariate analyses did not include other backyard sampled species, these being retained for descriptive reporting only. Production system was used as a key stratification variable to compare intensive and extensive poultry systems. County identifiers were harmonized using two-letter ISO abbreviations, and age was grouped into five categories: ≤36, 37–52, 53–78, 79–104, and 105–156 weeks.

#### 2.3.1. Descriptive Statistics

Seroprevalence was calculated as the proportion of positive samples for each serological outcome (MAT, ≥1:6, ≥1:12, ≥1:24, ≥1:48) stratified by production system, county, locality, age group, cat/mouse presence and flock size (extensive system only). Prevalence was expressed as percentages with 95% confidence intervals.

Additionally, a household cross-species seropositivity check was attempted for households with chickens and other avian backyard species, albeit to a limited degree due to the low sample sizes for these species.

#### 2.3.2. Bivariate Analysis

In the combined chicken-only dataset (*n* = 1880), each serological outcome (MAT, ≥1:6, ≥1:12, ≥1:24, and ≥1:48) was tested against the production system, county, locality, continuous age in weeks, and age group. Pearson’s chi-square test (χ^2^) was used for categorical variables, with Fisher’s exact test substituted for 2 × 2 tables when expected cell frequencies were <5. The Mann–Whitney U test was used for continuous age comparisons between seropositive and seronegative birds, and the Kruskal–Wallis H test was used for age-group comparisons. Cramér’s V was calculated as the effect size measure for chi-square tests and interpreted as: <0.10 negligible, 0.10–0.30 small-to-moderate, 0.30–0.50 moderate-to-strong, and >0.50 strong.

The family-wise error rate was controlled using Bonferroni correction, while the combined chicken-only full-inference framework included 25 tests (5 outcomes × 5 factors; α_corrected = 0.05/25 = 0.0020). A directly measured ground-truth sensitivity framework retained only the MAT, ≥1:24, and ≥1:48, resulting in 15 tests (3 outcomes × 5 factors; α_corrected = 0.05/15 = 0.0033). For the extensive backyard chicken-only subset, county, locality, cat presence, mouse presence, cat/mouse co-presence, age, and flock size were evaluated across the five serological outcomes; flock size was assessed using both Mann–Whitney U and Spearman’s rank correlation, yielding 40 exploratory tests (α_corrected = 0.05/40 = 0.0013).

#### 2.3.3. Multivariate Analysis

Binary logistic regression with L2 ridge regularization was used to model chicken seropositivity while reducing coefficient instability from sparse counties, localities, and quasi-complete separation. Species was not included as a predictor in the formal models because the inferential dataset contained only one normalized species category. Categorical predictors (county and locality) were one-hot encoded; production system was encoded as a binary variable, and continuous age in weeks was standardized before model fitting.

Core models were fitted for MAT, ≥1:24, and ≥1:48 outcomes using the county, production system, and age in weeks as predictors (16 features/model). A locality-expanded MAT sensitivity model was also fitted to assess whether finer geographic encoding changed the broad interpretation (41 encoded features), but locality coefficients were treated cautiously, with many locality cells being sparse.

Generalization performance was estimated by five-fold stratified cross-validation, and the training versus cross-validated accuracy is reported. Regression coefficients (β) and odds ratios (exp β) were interpreted as regularized associations rather than causal effects. Model definitions and performance metrics are provided in Table S4, and regularized coefficients are provided in Table S5.

#### 2.3.4. County-Overlap Sensitivity Analysis

Because intensive broilers were sampled only in Alba (AB), Mureș (MS), and Sibiu (SB), whereas backyard chickens were distributed across 12 non-overlapping counties, a within-county production-system comparison was not possible. As a pre-specified sensitivity analysis, we therefore (i) compared system-specific seroprevalence in the chicken-only dataset; (ii) repeated production-system chi-square tests for the MAT, ≥1:24, and ≥1:48; (iii) summarized backyard-chicken seroprevalence in Central Romania counties bordering the intensive sampling cluster (AG, BV, CJ, CV, MM, VL); and (iv) report crude odds ratios alongside the county-adjusted logistic models (Table S9). These analyses tested whether the production-system direction persisted when geography was partially aligned, but they did not remove confounding among age, system, and location.

## 3. Results

### 3.1. Descriptive Statistics

**Figure 1:**
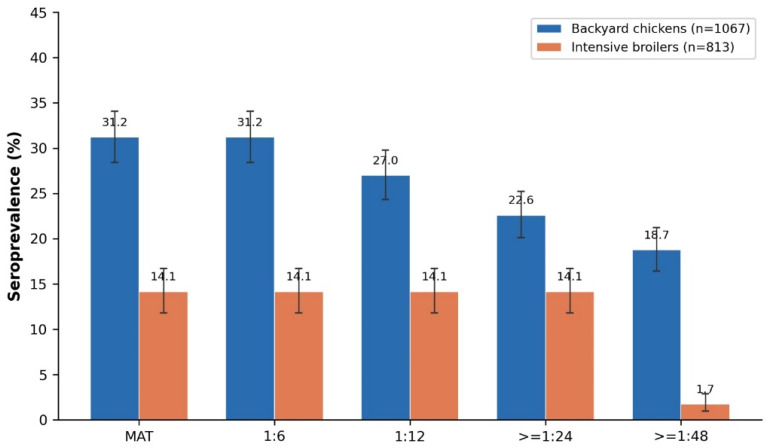
Chicken-only seroprevalence of *T. gondii* by production system across dilution thresholds. Grouped bar chart comparing MAT seroprevalence in extensive (*n* = 1067) vs. intensive (*n* = 813) production systems at MAT + four dilution cut-offs (MAT, ≥1:6, ≥1:12, ≥1:24, ≥1:48). Error bars represent 95% Clopper–Pearson exact confidence intervals. MAT = any positive result; 1:6/1:12 inferred for intensive from 1:24 cascade (Table S2).

A total of 1940 domestic birds from two production systems were tested for anti-*T. gondii* antibodies by the MAT at serial dilutions (Table 1). Due to the intensive broilers’ and backyard birds’ differences in age structure, management, and county distribution, seroprevalence is reported by production system rather than as a single pooled estimate. In the chicken-only inferential subset (*n* = 1880), the MAT seroprevalence was 31.2% (22.4% at 1:24 cut-off) in backyard chickens and 14.1% in intensive broilers, with a stark ≥1:48 contrast between the two systems (18.7% versus 1.7%) (Table 1, Figure 1). The full descriptive dataset, including non-chicken species, is featured in Table S8.

**Figure 2:**
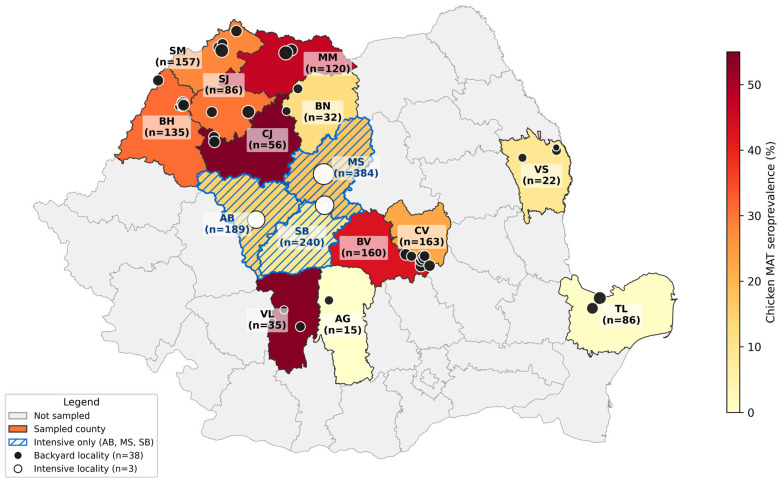
County and locality choropleth of MAT seroprevalence.

Backyard chickens displayed higher seroprevalence than intensive broilers at all thresholds (production-system comparison *p* < 0.001). The age-group seroprevalence among the chickens peaked at 37–52 weeks (37.1%) and 105–156 weeks (33.3%). The age-group rows are descriptive because all 42-day broilers fell in the ≤36 weeks stratum and were structurally confounded with intensive production. The county-level MAT seroprevalence in the chickens is shown in Figure 2.

### 3.2. Association and Regression Analysis

#### 3.2.1. Combined Database

In the combined chicken-only dataset (*n* = 1880), 25 bivariate tests were performed across five serological outcomes and five factors (production system, county, locality, age in weeks, and age group). All 25 tests were significant at alpha = 0.05, and 24/25 survived Bonferroni correction (alpha_corrected = 0.0020). The single Bonferroni exception was ≥1:24 × age_weeks (Mann–Whitney U = 250,523.5, *p* = 0.017), consistent with weaker age separation at the intermediate dilution threshold.

In unadjusted bivariate tests, locality and county showed the largest effect sizes (Table 2, locality V = 0.42 for the MAT and V = 0.45 for ≥1:48). Production system was also significantly associated, with its effect size increasing at higher dilution thresholds (V = 0.20 for the MAT; V = 0.26 for ≥1:48). At the alternative shared ≥1:24 cut-off, the production-system association remained significant but was weaker (V = 0.11; Table 2), consistent with exclusion of backyard birds reactive only at 1:6 or 1:12. All intensive broilers were 42 days old, whereas backyard chickens ranged from 24 to 156+ weeks; age and production system were therefore completely confounded in the combined dataset and could not be interpreted as independent effects without age-matched cross-system sampling. Since intensive broilers were sampled only in Alba, Mureș, and Sibiu, whereas backyard chickens were distributed across 12 non-overlapping counties (Figure 2), geographic heterogeneity also overlapped with the production system.

For intensive broilers, dilutions 1:6 and 1:12 were inferred from directly measured 1:24 results using the dilution cascade (Table S2). The alternative ground-truth framework restricted to directly measured dilutions (MAT, ≥1:24, ≥1:48) confirmed the same production-system direction at the shared ≥1:24 cut-off (22.6% backyard vs. 14.1% intensive; *p* < 0.001), with the absolute gap narrower than that for any positive MAT result (31.2% vs. 14.1%) but still significant after Bonferroni correction. No county included both backyard and intensive chickens; county-overlap sensitivity analyses confirmed the same production-system direction when system-specific seroprevalence was compared, production-system chi-square tests were repeated, and backyard chickens in Central Romania border counties were examined (MAT 38.6% vs. intensive 14.1%; crude OR for intensive vs. backyard = 0.36; Table S9). These checks did not eliminate age–system–geography confounding but show that the contrast was not driven solely by the widest geographic comparison.

**Figure 3:**
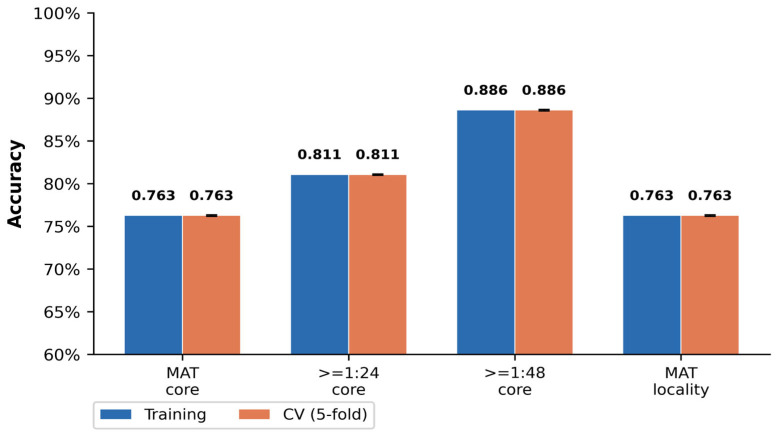
Cross-validation performance by model specification. Training and five-fold cross-validation accuracy for the MAT, ≥1:24, and ≥1:48 core models, plus the locality-expanded MAT sensitivity model.

**Figure 4:**
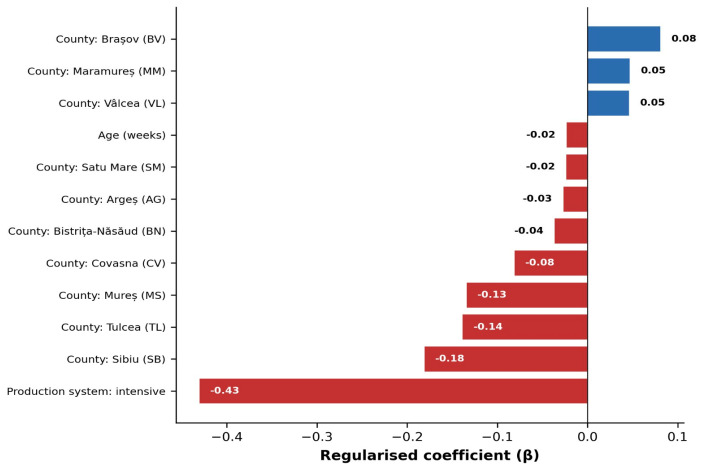
Largest regularized coefficients. A horizontal bar chart of the largest absolute regularized coefficients in the chicken-only MAT model. Coefficients are shrinkage estimates and should be interpreted as ranked associations rather than causal effects.

The core models (MAT core, ≥1:24 core, ≥1:48) achieved training accuracies of 0.762–0.886± 0.001. These logistic regression models, including county, production system, and age in weeks, were fitted as an explanatory check rather than as predictive tools (Figure 3; Table S4). After county and age were accounted for, intensive production was the variable most strongly associated with lower odds of seropositivity (β = −0.43, OR = 0.65 for the MAT; β = −0.57, OR = 0.56 for ≥1:48) (Figure 4). County effects were smaller in the adjusted models than in unadjusted bivariate tests, consistent with overlap between geographic sampling and production systems rather than evidence that location is unimportant. Adding farm-level locality labels did not improve model performance beyond the simpler specification, indicating that finer geographic detail did not add stable information once the county and production system were included (model accuracy detailed in Table S4).

#### 3.2.2. Intensive System

The intensive subset contained 813 broiler chickens of uniform age (42 days); therefore, species and age could not be evaluated within this partition. Variation was limited to the farm/locality and county. Locality/county associations with ≥1:48 were the only tests to survive Bonferroni correction, reflecting inter-farm variation in contamination even within industrial settings. Logistic regression on the intensive subsample used only locality/county dummies as predictors. The training accuracy was 0.859 for the MAT and 0.983 for ≥1:48. The extreme accuracy at ≥1:48 reflects severe class imbalance (only 14/813 positives). Geography captures inter-farm differences in seropositivity, while the very low ≥1:48 prevalence (1.7%) makes high-titer modeling sensitive to class imbalance (Supplementary Table S5).

#### 3.2.3. Extensive System

Within the extensive backyard chicken subset (*n* = 1067), 15/40 exploratory bivariate tests were significant at alpha = 0.05, and 14/40 survived Bonferroni correction (alpha_corrected = 0.0013) (Table S3). Significant associations were driven primarily by locality, county, and age. Cat presence, mouse presence, cat/mouse co-presence, and flock size did not provide stable evidence of associations after correction. The limited variability of cat and mouse presence across the sampled farms (90.1% with cats, 91.5% with mice) prevented statistical assessment.

Extensive chicken-only logistic models produced cross-validated accuracies of 0.688 for the MAT, 0.774 for ≥1:24, and 0.813 for ≥1:48. These values are useful for model comparison but should not be overinterpreted as indicating strong predictive performance, particularly at higher dilutions (Table S6).

#### 3.2.4. Descriptive Cross-Species Household Check

Other backyard avian species were too sparse for formal species-level inference (Table 1). A household-level exploratory check among mixed chicken/non-chicken backyard households found 17 locality–household keys containing chickens and at least one other species, with 49 non-chicken records. Household-level chicken positivity was not significantly associated with other backyard species positivity (Fisher’s exact *p* = 0.637; Spearman’s rho for household prevalence = 0.041, *p* = 0.875) (Table S7). These data support, at most, a descriptive co-exposure observation and should not be used to claim that chicken seropositivity predicts turkey, duck, guinea fowl, or pigeon seropositivity.

## 4. Discussion

### 4.1. Production System Contrast

Backyard chickens had higher MAT seroprevalence than intensive broilers (31.2% vs. 14.1%), consistent with global comparisons of free-range and confined poultry [2,20,21]. Under the alternative shared ≥1:24 cut-off, the contrast narrowed to 22.6% versus 14.1% but remained significant (Table 1), indicating that part of the MAT-level gap reflects back-yard birds reactive only at lower dilutions that were not tested in broilers. This marked difference is consistent with the known transmission dynamics of *T. gondii*, as backyard chickens have continuous access to soil contaminated with oocysts shed by peridomestic cats [4,5]. Comparable feeding-ecology gradients in wild birds (18% in rooks vs. 0.5% in sparrows in the Czech Republic) support interpreting ground-contact patterns as exposure-relevant rather than taxonomic artefacts [32]. While biologically compatible, the MAT detects prior exposure only and cannot by itself (without a bioassay or molecular data) [29,33] quantify environmental contamination or prove a causal effect of management. Turkeys, ducks, guinea fowl, and pigeons were retained descriptively only (Table S8); their small sample sizes precluded stable species-level inference, so formal comparisons used the normalized chicken-only subset throughout.

Industrial biosecurity measures in intensive operations substantially limit this exposure route [10,11], although the 14.1% intensive prevalence shows that exposure is reduced rather than absent. The system contrast widened 11-fold at ≥1:48 (Table 1), indicating that high-titer responses were concentrated in backyard chickens. However, due to MAT limitations and age confounding (all intensive broilers were of 42 days of age, while backyard chickens had variability) this gap cannot be attributed exclusively to production systems without age-matched cross-system sampling. Overall, our results are consistent with the meta-analytic OR of approximately 2–3× reported in systematic reviews comparing free-range to confined poultry globally [2,20].

### 4.2. Romania Within International Context

This study provides the first avian intensive-system *T. gondii* seroprevalence baseline for Romania, alongside updating that of backyard chickens. While Dubey et al. [24] provided a comprehensive review of human and animal toxoplasmosis in Romania (human seroprevalence 36–70% depending on county, age, and method), domestic birds were largely absent.

Cross-study comparisons are limited by the diagnostic method (MAT vs. ELISA/IFAT) [29,30]. In the present study, the MAT was chosen to screen the multi-species backyard cohort with one host-independent panel, using a validated antigen from the French National Reference Centre [28,31], whereas our previous study [25] applied an in-house chicken ELISA in the preceding extensive survey from the same program (with a reported 38.6% seroprevalence). In China, the MAT seroprevalence in market-sold chickens is 5.8–7.3%, with caged birds at roughly half the prevalence of free-range counterparts [14,15]. National syntheses report a pooled chicken seroprevalence near 20% with regional heterogeneity [16]. Global meta-analyses place intensive systems at 5–8% and backyard operations at 20–50% [21]. Similar outdoor-exposure signals appear in Australian commercial free-range chickens (~44%) [13] and Libyan free-range flocks (39.2% in older birds by ELISA) [17]. Together, these patterns support interpreting Romania’s 14.1% intensive estimate as being above many industrial benchmarks worldwide [22], while both 31.2% (any positive MAT result) and 22% (at a shared 1:24% cut-off) fall within the expected European backyard range [10,11]. Physical exclusion from oocyst-contaminated soil remains the primary protective factor, and Romania’s intensive prevalence appears intermediate between the near-zero values for highly industrialized settings and moderate values for European systems with residual environmental exposure [2,21].

### 4.3. Geographic Heterogeneity and Overlap with Production System

In unadjusted analyses, geographic administrative descriptors showed the strongest categorical associations with seropositivity (Table 2). Locality (Cramér’s V = 0.42) exceeded county (V = 0.32), consistent with finer-scale environmental heterogeneity within counties. At the county level in the same inferential dataset, the descriptive MAT seroprevalence was highest in Cluj (CJ 55.4%), Vâlcea (VL 54.3%), and Maramureș (MM 46.7%) while those for Tulcea (TL 1.2%) and Vaslui (VS 9.1%) were among the lowest (Figure 2).

These patterns must be interpreted alongside structural confounding. Intensive broilers were sampled only in Alba (AB), Mureș (MS), and Sibiu (SB), whereas backyard chickens were distributed across 12 non-overlapping counties. No county included both systems, so county-level contrasts overlapped with production-system contrasts (Table S9). Pre-specified sensitivity analyses comparing system-specific seroprevalence, repeating production-system chi-square tests, and summarizing backyard chickens in Central Romania counties bordering the intensive cluster (MAT 38.6%, *n* = 549) preserved the same direction of association (backyard > intensive) but did not remove age–system–geography confounding.

The county choropleth (Figure 2) therefore provides a descriptive spatial context for surveillance prioritization rather than formal spatial inference. Since household coordinates were unavailable and sample sizes were uneven across counties, a county-level Moran’s I would largely recapitulate the categorical heterogeneity (Table 2). Variation among counties may reflect unmeasured differences in cat density, soil conditions, farm hygiene, or husbandry practices; however, these variables were not measured in the present study [2,3]. Counties with descriptive MAT seroprevalence above ~45% may warrant targeted follow-up, including molecular typing and age-matched sampling, rather than uniform nationwide intervention.

### 4.4. The Cat/Mouse Paradox: Ubiquity Masking Causality

An initially counterintuitive finding was the lack of a detectable statistical association between cat or mouse presence and MAT seroprevalence in the extensive system, despite cats being the definitive host of *T. gondii* [4,5]. Because of their near-universal presence (90.1% and 91.5% of households) this exposure was functionally constant and could not be evaluated as a risk factor under the present design [2,3]. Oocyst contamination also depends on the feline age structure and shedding dynamics, as cats typically shed oocysts after primary infection rather than continuously [4,5]. This study recorded only binary cat presence and not cat age, density, or shedding timing, further limiting interpretation of the null household-level association.

Similarly, flock size showed no association with seroprevalence (Spearman’s ρ = −0.002, *p* = 0.94), supporting the interpretation that farm-level environmental variation was more informative than the measured household-level husbandry variables within the extensive system.

### 4.5. Dilution-Stratified Risk Architecture

Seroprevalence declined monotonically across the MAT dilution thresholds in both production systems (Table 1; Figure 1), with positivity falling at each successive dilution step rather than collapsing to a single exposed/unexposed dichotomy. Among MAT-seropositive chickens, only approximately half maintained reactivity at ≥1:48, reflecting titer attrition across the dilution series rather than a direct readout of infection status; the MAT cannot confirm whether persisting antibodies reflect viable tissue cysts, resolved exposure, or active infection without a bioassay or molecular follow-up [2,29].

In intensive broilers, 1:6 and 1:12 reactivity was inferred from directly measured 1:24 results using a dilution cascade validated in the extensive dataset (Table S2). The resulting four dilution columns were perfectly collinear in this subsample, reducing model stability without altering the directional dilution pattern. These inferred thresholds improve descriptive comparability in Figure 1 but are not independent measurements and should be interpreted as cascade-derived comparators rather than separately observed outcomes.

Due to dilution sampling asymmetry across the production systems a shared ≥1:24 threshold was used to assess common dilution seroprevalence. The backyard chicken value was 22.6%, versus 14.1% in intensive broilers. The absolute system gap therefore narrowed relative to the any-positive-MAT contrast (31.2% vs. 14.1%), but the backyard > intensive direction remained significant. The ≥1:48 contrast (18.7% vs. 1.7%), for which both systems were tested directly, was unchanged by cascade inference and remained the steepest titer disparity. Thus, asymmetric low-dilution panels may inflate MAT-level differences, yet they do not create the production-system pattern observed at shared measured thresholds.

### 4.6. One Health, Adjusted Associations, and Public Health Implications

County- and age-adjusted ridge logistic models confirmed the bivariate pattern of the production system and geography dominating adjusted associations, while adding locality did not improve stability (Table S4). However, coefficients should be read as explanatory summaries, not causal or predictive estimates.

From a One Health perspective, domestic poultry are intermediate hosts and potential sources of infection for humans and predators [2]. Backyard chickens integrate local soil oocyst exposure through ground foraging and can signal settings where humans might encounter contaminated environments or tissues, while MAT serology documents exposure but cannot quantify human risk [2,7,14,34,35].

Zoonotic relevance must be interpreted cautiously. Seropositivity indicates prior exposure, not the confirmed presence of viable tissue cysts in edible meat [2,29,31]. In rural Romania, informal backyard slaughter and undercooked organ consumption represent a plausible exposure route [23]. On the other hand, the MAT titer, including higher ≥1:48 seroprevalence in backyard birds, cannot be equated with foodborne infectivity without tissue-based confirmation [2,4]. Targeted follow-up, including genotyping, is warranted in counties with high descriptive MAT seroprevalence, given the unknown genotype distribution in domestic poultry despite Type III circulation in wild corvids [6,26,27]. This could also be expanded to counties reporting high prevalence in other backyard or wild birds, particularly with atypical genotypes [8,9].

From an economic standpoint, subclinical infections may impair weight gain and feed conversion, with estimated annual losses of $5–15 million in regions with significant backyard livestock sectors (Brazil) [36]; however, local estimates for Romania might differ. Overall, Romania’s extensive-system prevalence appears consistent with the broader Central–Eastern European patterns, rather than a national outlier [21].

### 4.7. Limitations

Several limitations warrant acknowledgment. Cross-sectional MAT serology cannot establish causality, genotype, or cyst viability. Age, production system, and geography were structurally confounded, while cat and mouse presence was recorded as binary only. Lower dilutions in intensive broilers were inferred from ≥1:24 results (validated in the extensive dataset; Table S2). Same-age, same-county sampling is needed for stronger causal inferences.

## 5. Conclusions

This study provides the first structured comparison of *T. gondii* MAT seroprevalence between backyard and intensive broiler chickens in Romania. Backyard chickens had higher seroprevalence than intensive broilers (31.2% vs. 14.1% MAT; 18.7% vs. 1.7% at ≥1:48), but age, geography, and production system were confounded, and no county included both systems. The MAT indicates prior exposure only. Molecular typing, age-matched sampling, and integration with slaughter and consumption practices are priorities for clarifying zoonotic relevance.

Overall, the findings support backyard chickens as practical sentinels of environmental exposure to *T. gondii* and suggest that intensive production reduces, but does not eliminate, exposure.

## Figures and Tables

**Table 1:** MAT seroprevalence and dilution-specific positivity in chickens by production system and age group (chicken-only inferential subset).

Variable	Category	N	MAT%	95% CI	1:6 (%)	1:12 (%)	1:24 (%)	1:48 (%)	*p*
Production system (FS)	Backyard	1067	31.2	28.4–34.1	31.2	27.0	22.6	18.7	<0.001
	Intensive	813	14.1	11.8–16.7	14.1	14.1	14.1	1.7	<0.001
Age group (FS)	≤36 w	813	14.1	11.8–16.7	14.1	14.1	14.1	1.7	-
	37–52 w	501	37.1	32.9–41.5	37.1	32.7	27.9	22.4	-
	53–78 w	262	31.3	25.7–37.3	31.3	26.3	21.4	18.3	-
	79–104 w	199	15.1	10.4–20.8	15.1	12.6	10.6	10.1	-
	105–156 w	105	33.3	24.4–43.2	33.3	28.6	22.9	19.0	-

*p*-value applies to the production-system chi-square comparison only; age-group rows are descriptive (-). MAT = Modified Agglutination Test (any positive titer). 95% CI = Clopper–Pearson exact binomial confidence interval for MAT%. The ≤36 weeks stratum comprised only 42-day intensive broilers and was structurally confounded with the production system.

**Table 2:** Core bivariate associations in the combined dataset (*n* = 1880).

MAT Dilution	Statistic	Locality χ^2^	County χ^2^	Production System χ^2^	Age (Weeks) M-W U	Age Group K-W H
1:24	*p* value	<0.0001	<0.0001	<0.0001	0.017	<0.0001
	Effect	V = 0.34	V = 0.25	V = 0.11	r = 0.08	-
1:48	*p* value	<0.0001	<0.0001	<0.0001	<0.0001	<0.0001
	Effect	V = 0.45	V = 0.37	V = 0.26	r = 0.35	-
Any	*p* value	<0.0001	<0.0001	<0.0001	<0.0001	<0.0001
	Effect	V = 0.42	V = 0.32	V = 0.2	r = 0.17	-

M-W U = Mann–Whitney U test; K-W H = Kruskal–Wallis H test; χ^2^—chi-square test; V = Cramér’s V; r = Mann–Whitney rank-biserial correlation. Bonferroni correction: α_corrected = 0.05/25 = 0.002. Results for 1:6 and 1:12 omitted for brevity (identical to MAT for 1:6; intermediate values for 1:12—Table S1).

## Data Availability

The data presented in this study are available upon request from the corresponding author due to confidentiality and institutional restrictions.

## Supplementary Materials

The following supporting information can be downloaded at https://www.mdpi.com/article/10.3390/ani16152313/s1, Table S1: Bivariate test results—poultry combined; Table S2: Bivariate test results—poultry ground truth; Table S3: Bivariate test results—extensive chickens; Table S4: Poultry logistic model summary; Table S5: Poultry logistic coefficients; Table S6: Extensive chicken logistic coefficients; Table S7: Household cross-species check; Table S8: Descriptive prevalence for all species; Table S9: County-overlap sensitivity.

## Abbreviations

The following abbreviations are used in this manuscript:

MAT	Modified Agglutination Test
PCR	Polymerase Chain Reaction
95% CI	95% confidence interval
